# The Effect of Heat Treatment on the Emission Color of P-Doped Ca_2_SiO_4_ Phosphor

**DOI:** 10.3390/ma10091000

**Published:** 2017-08-26

**Authors:** Hiromi Nakano, Konatsu Kamimoto, Nobuyuki Yokoyama, Koichiro Fukuda

**Affiliations:** 1 Cooperative Research Facility Center, Toyohashi University of Technology, Toyohashi 441-8580, Japan; 2 Department of Environmental and Life Sciences, Toyohashi University of Technology, Toyohashi 441-8580, Japan; k163413@edu.tut.ac.jp (K.K.); john.n.yokoyan@gmail.com (N.Y.); 3 Department of Materials Science and Engineering, Nagoya Institute of Technology, Nagoya 466-8555, Japan; fukuda.koichiro@nitech.ac.jp

**Keywords:** optical materials, phase transition, crystal structure, transmission electron microscopy

## Abstract

In a series of (Ca_2–*x*/2–*y*_Eu*_y_*□*_x_*_/2_)(Si_1–*x*_P*_x_*)O_4_ (*x* = 0.06, 0.02 ≤ *y* ≤0.5), various color-emitting phosphors were successfully synthesized by a solid-state reaction. These phosphors were characterized by photoluminescence (PL) spectroscopy, X-ray powder diffractometry, transmission electron microscopy, and X-ray absorption fine structure spectroscopy. We evaluated the effect of heat treatment on PL properties with various annealing temperatures at 1373–1773 K for 4 h before/after reduction treatment from Eu^3+^ to Eu^2+^. In the red-emitting (Ca_1.95_Eu^3+^_0.02_□_0.03_)(Si_0.94_P_0.06_)O_4+δ_ phosphor, the highest PL intensity exhibited when it was annealed at 1773 K. On the other hand, in the green-emitting (Ca_1.95_Eu^2+^_0.02_□_0.03_)(Si_0.94_P_0.06_)O_4_ phosphor, the highest PL intensity was realized when it was annealed at 1473 K and consequently treated under a reductive atmosphere. With increasing annealing temperature, the emission peak wavelength steadily decreased. Furthermore, with increasing Eu^2+^ content, the emission peak wavelength increased, with the color of emitting light becoming yellowish. Thus, the PL properties of the phosphors were affected by both the structural change from β to α’_L_, which occurred by heat treatment, and the amount of doped Eu ions.

## 1. Introduction

Rare-earth-doped dicalcium silicate (Ca_2_SiO_4_, C_2_S) phosphors have been investigated by several researchers for application to white light-emitting diodes (LEDs) [1,2,3,4,5]. Two types of phosphors have been reported so far: Eu^3+^-doped and Eu^2+^-doped C_2_S phosphors. The former phosphors are characterized by the emission of red light due to the transition of ^5^D_0_-^7^F_2_ for the Eu^3+^ ion [3]. Thus, the luminescence originates from the 4f-4f dipole transitions of the Eu^3+^ ion, and hence the wavelength of the emission is almost the same among the various phosphors with different host materials. For example, a group of A_2_SiO_4_: Eu^3+^ (A = Ca, Sr, Ba) phosphors showed similar emission spectra at around the maximum wavelength of 620 nm [4]. On the other hand, Eu^2+^-doped C_2_S phosphors are generally useful for a wide range of applications, because their luminescent colors, due to the f-d transition of the Eu^2+^ ion, are tunable by the crystal structures and/or compositions of the host materials. Recently, a crystal-site engineering technique was reported, which enabled us to customize the luminescent colors of C_2_S:Eu^2+^ phosphors [5]. 

A series of structural studies of P-doped C_2_S crystals has demonstrated that the incorporation of P most effectively stabilized the high-temperature phases of C_2_S [6]. Hence, the authors present the idea of utilizing the P-doped C_2_S as the host material of Eu^2+^-activated phosphors. In a previous study, the relationship between the photoluminescence (PL) intensities and crystal structures of P-doped C_2_S phosphors was reported [7]. The doping of P ions in the green-emitting C_2_S:Eu^2+^ phosphors were found to effectively increase the PL intensities. The polymorphs of C_2_S established are, in the order of increasing temperature, γ (orthorhombic), β (monoclinic), α’_L_ (orthorhombic), α’_H_ (orthorhombic), and α (trigonal) [8]. The phase transition temperatures are, during the heating process, 963 K for β to α’_L_, 1433 K for α’_L_ to α’_H_, and 1698 K for α’_H_ to α. The β to α’_L_ phase transition of the cooling of doped C_2_S has been reported to be thermoelastic martensitic [9], hence the stabilized phases at ambient temperature systematically changed from β, β+α’_L_ to α’_L_ with increasing concentrations of foreign ions such as Sr^2+^, Ba^2+^, and/or P^5+^ in C_2_S [10,11]. Thus, the change of crystal structures by heat treatment must be potentially able to improve the photoluminescence (PL) properties of the phosphors. In fact, we have succeeded in the further improvement of the PL intensities by the annealing of the green-emitting P-doped C_2_S phosphor [12]. 

In the present study, we successfully synthesized P-doped C_2_S phosphors showing various emission colors (red, green, and yellow) by a solid-state reaction. The PL intensities were compared between the phosphors with different activators of Eu^3+^ or Eu^2+^ ions that were annealed at 1373–1773 K. We also discussed the relationship between the PL intensities and crystal structures.

## 2. Results and Discussion

### 2.1. Red-Emitting P-Doped C_2_S:Eu^3+^ Phosphor

In a previous study, we prepared a series of (Ca_1.98–*x*/2_Eu^2+^_0.02_□*_x_*_/2_)(Si_1–*x*_P*_x_*)O_4_ (P-doped C_2_S:Eu^2+^) phosphors with 0 ≤ *x* ≤ 0.20 (*y* = 0.02), and investigated the effect of P^5+^-ion doping on the PL properties. Here, the box in the chemical formula represents vacancies according to a previous paper [6]. The P-doped C_2_S:Eu^2+^ phosphor with *x* = 0.06 exhibited the highest PL intensity among those with 0 ≤ *x* ≤ 0.20 [12]. 

In the present study, we prepared the Eu^3+^-activated red-emitting phosphor with a chemical formula of (Ca_1.9__5_Eu^3+^_0.02_□_0.03_)(Si_0.94_P_0.06_)O_4__+δ_, and focused on the effect of annealing on its PL property.

**Figure 1:**
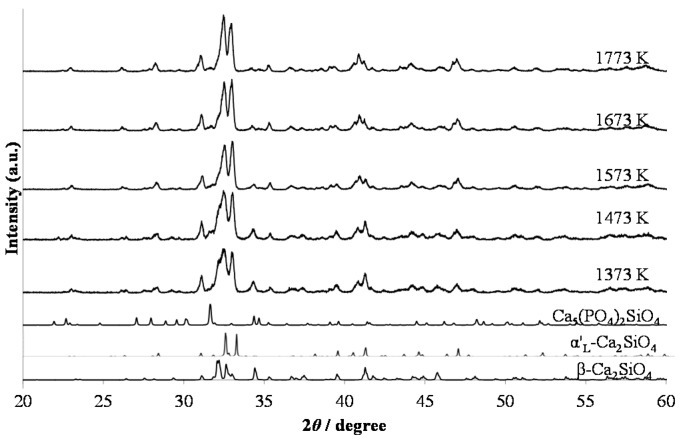
XRD patterns of (Ca_1.95_Eu^3+^_0.02_□_0.03_)(Si_0.94_P_0.06_)O_4+δ_ annealed at various temperatures from 1373 K to 1773 K for 4 h.

**Figure 2:**
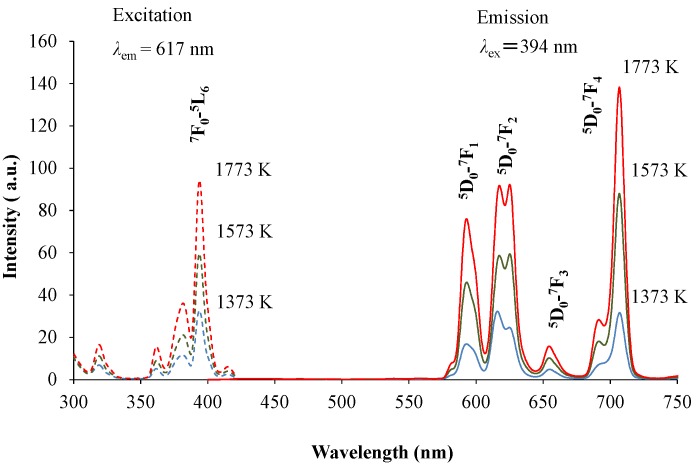
Emission and excitation spectra of (Ca_1.95_Eu^3+^_0.02_□_0.03_)(Si_0.94_P_0.06_)O_4+δ_ annealed at 1373 K, 1573 K, and 1773 K.

**Figure 3:**
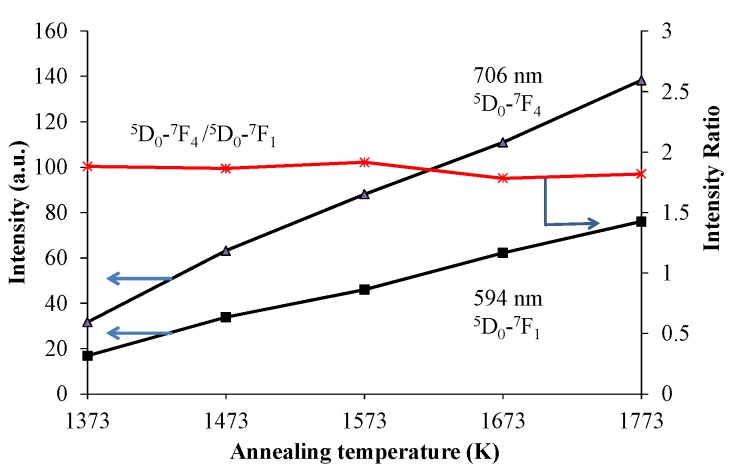
The emission intensities of (Ca_1.95_Eu^3+^_0.02_□_0.03_)(Si_0.94_P_0.06_)O_4+δ_ at 706 nm and at 594 nm excited by 394 nm. The ratio of I_706_ nm/I_594_ nm is also plotted.

Figure 1 shows the X-ray diffraction (XRD) patterns of the red-emitting phosphor annealed at various temperatures from 1373 K to 1773 K for 4 h. With increasing annealing temperature, the relative amount of the α’_L_ phase with respect to the β phase increased. This is caused by the phase transition from β to α’_L_ during annealing. Figure 2 shows the emission and excitation spectra of the red-emitting phosphors annealed at 1373 K, 1573 K, and 1773 K. Red light emission of the Eu^3+^-activated phosphors were observed at an excitation wavelength of 394 nm due to the intraconfigurational ^7^F_0_-^5^L_6_ transition. We observed the highest PL intensity for the specimen that was annealed at 1773 K. The sharp and strong emission peaks, induced by the transitions in the Eu^3+^ ion, appeared at around 594 nm (^5^D_0_-^7^F_1_, magnetic-dipole), 625 nm (^5^D_0_-^7^F_2_, electric-dipole), and 706 nm (^5^D_0_-^7^F_4_, electric-dipole). We plotted the emission intensity at 594 nm (=I_594_ nm) and that at 706 nm (=I_706_ nm) with excitation by 394 nm in Figure 3. The intensity ratios of I_706_ nm/I_594_ nm (=^5^D_0_-^7^F_4_/^5^D_0_-^7^F_1_) with different annealing temperatures are also plotted in the figure. Both I_594_ nm- and I_706 nm_-values steadily increased with increasing annealing temperature, while there was no significant change in the intensity ratio of I_706_ nm/I_594_ nm. The electric-dipole transitions, which is the f-f transition from ^5^D_0_ to ^7^F_2,4_, are, according to the theory of selection rule [13], affected by the site symmetry of the crystal fields around the Eu^3+^ ion. From the present results, the Eu^3+^ ion would be in the same Ca site, although the site environment of the Eu^3+^ ion must be changed from the β phase to the α’_L_ phase. Interestingly, in our previous paper, bright red emission due to hypersensitive ^5^D_0_-^7^F_2_ (electric-dipole) transition and very small ^5^D_0_-^7^F_4_ and ^5^D_0_-^7^F_1_ transitions were observed under the ultraviolet irradiation of 541 nm or 398 nm to Li_1.11_Ta_0.89_Ti_0.11_O_3_ phosphor [14]. The eccentricity of the Eu^3+^ positions in [(Li, Eu)O_12_] polyhedron thus seems to be closely related to the PL efficiency of the doped lithium tantalate phosphors [15].

### 2.2. Green-Emitting P-Doped C_2_S:Eu^2+^ Phosphor

**Figure 4:**
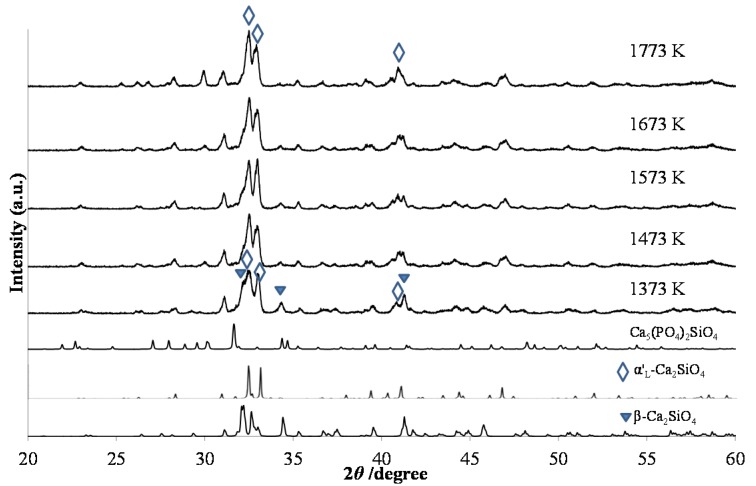
XRD patterns of (Ca_1.95_Eu^2+^_0.02_□_0.03_)(Si_0.94_P_0.06_)O_4_ annealed at various temperatures from 1373 K to 1773 and subsequent heating under 97% Ar–3% H_2_.

**Figure 5:**
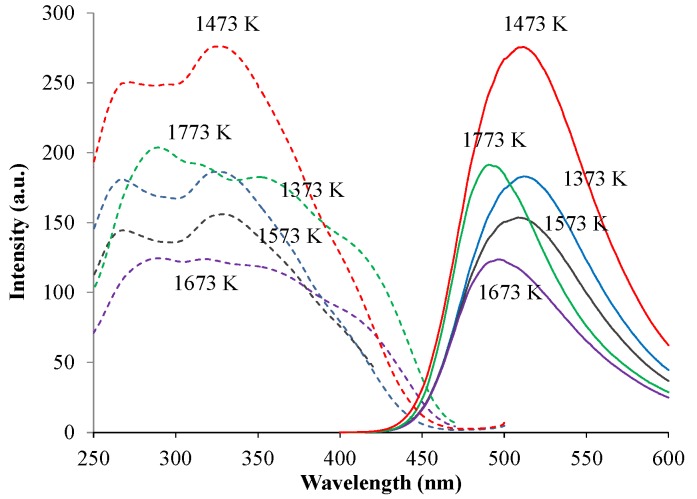
Relationship between photoluminescence (PL) intensity and annealing temperature in (Ca_1.95_Eu^2+^_0.02_□_0.03_)(Si_0.94_P_0.06_)O_4_ phosphors.

The phase compositions of green-emitting (Ca_1.95_Eu^2+^_0.02_□_0.03_)(Si_0.94_P_0.06_)O_4_ phosphors were, as shown in Figure 4, different among those with different annealing temperatures. When comparing the phase compositions between the two types of phosphors, (Ca_1.95_Eu^2+^_0.02_□_0.03_)(Si_0.94_P_0.06_)O_4_ and (Ca_1.95_Eu^3+^_0.02_□_0.03_)(Si_0.94_P_0.06_)O_4+δ_, at the same annealing temperatures, they were almost equal to each other. Figure 5 shows the relationship between the PL intensity and annealing temperature in green-emitting (Ca_1.95_Eu^2+^_0.02_□_0.03_)(Si_0.94_P_0.06_)O_4_ phosphors. The broad excitation spectra were composed of the four bands at 310 nm, 340 nm, 360 nm, and 400 nm, each of which could be attributed to the location of an Eu^2+^ ion in the crystal structures of the β- and α’_L_-phases [16,17]. The differences in peak shapes of the excitation spectra are caused by the differences in crystal structures of the host materials. The emission or excitation wavelengths were measured by monitoring them at the maximum wavelengths, as shown in Table 1. The emission peak wavelength decreased with increasing annealing temperature. This is caused by the expansion of Eu–O bond lengths by phase transition [17], because the 4f-5d transition of the Eu^2+^ ion must be closely related to the crystal field. We observed the highest PL intensity for the specimen annealed at 1473 K, of which the phase composition was both β and α’_L_. 

**Figure 6:**
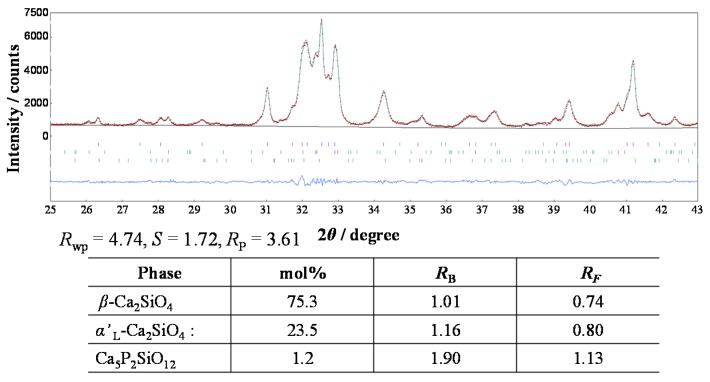
Observed profile intensities (red symbol: t), fitted patterns (green solid lines), and difference curves (blue solid lines in lower part of diagrams) obtained from the (Ca_1.95_Eu^2+^_0.02_□_0.03_)(Si_0.94_P_0.06_)O_4_ annealed at 1473 K.

We investigated the profile intensity data of the phosphor sample annealed at 1473 K by the Rietveld method, and determined the precise phase composition. The structural parameters were taken from those reported by Jost et al. [18] for β-C_2_S, Udagawa et al. [19] for α’_L_-C_2_S, and Dickens and Brown [20] for Ca_5_P_2_SiO_12_. The sample was found to be composed of both the β- and α’_L_-phases with a small amount of Ca_5_P_2_SiO_12_ as the impurity phase. The phase composition was determined to be 75.3 mol % β, 23.5 mol % α’_L_, and 1.2 mol % Ca_5_P_2_SiO_12_ (Figure 6), under the condition of each effective particle radii being 5 μm. Because the highest PL intensity for the present sample among the phosphors annealed at 1373–1673 K, the co-existence of the β- and α’_L_-phases would be essentially important for the enhancement of the PL intensity of green-emitting P-doped C_2_S:Eu^2+^ phosphors. This is because the coherent interphase boundaries between α’_L_ and β would store the strain energy, which would distort the crystal lattices and provide a favorable environment for the efficient PL emission of the Eu^2+^ ion.

**Figure 7:**
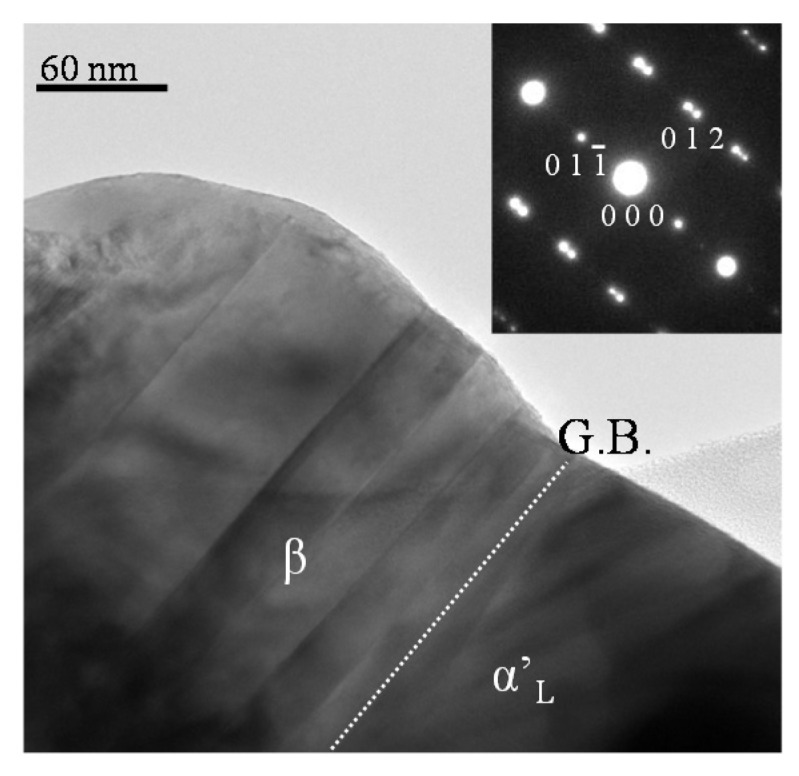
TEM image taken from the ($\bar{1}$00) and SAED pattern of (Ca_1.95_Eu^2+^_0.02_□_0.03_)(Si_0.94_P_0.06_)O_4_ phosphor annealed at 1473 K.

Figure 7 shows a transmission electron microscope (TEM) image of (Ca_1.95_Eu^2+^_0.02_□_0.03_)(Si_0.94_P_0.06_)O_4_ phosphor annealed at 1473 K taken from the ($\bar{1}$00), and the corresponding selected area electron diffraction (SAED) pattern. The relevant crystal grain was made up of the two regions of β and α’_L_. The β-phase region showed a pseudo-merohedral (polysynthetic) twin structure, with the splitting of reflections for the SAED pattern. The reflection indices are based on the β-phase lattice. The coherent grain boundary (depicted by G. B. in Figure 7) was parallel to the ($01\bar{1}$) plane of the β phase (*a* = 5.513, *b* = 6.758, *c* = 10.460 nm, β = 117.27 degrees) and the (001) plane of the α’_L_ phase (*a* = 20.470, *b* = 9. 390, *c* = 5.437 nm). We examined the XRD profile intensity data by the Rietveld method to find that the geometrical difference in the boundaries between ($01\bar{1}$) and (001) was as small as 0.023 nm. The coherent interphase boundaries between α’_L_ and β would store the strain energy, which would distort the crystal lattices and, consequently, provide a favorable environment for the efficient PL emission of the Eu^2+^ ion in the green-emitting phosphor.

**Figure 8:**
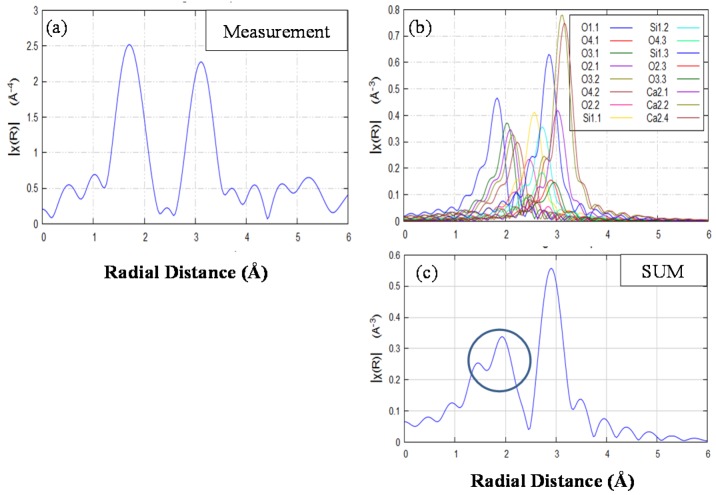
Radial structure function of (Ca_1.95_Eu^2+^_0.02_□_0.03_)(Si_0.94_P_0.06_)O_4_ phosphor annealed at 1473 K in (**a**). (**b**) Simulation spectra, in which the Eu ion is substituted in the Ca(1n) site in β-Ca_2_SiO_4_. (**c**) Calculated peaks as a sum of each peak shown in (**b**).

In order to determine the environment of the Eu ion, X-ray absorption fine structure (XAFS) spectra were obtained with fluorescence mode at room temperature. Figure 8 shows the radial structure function of (Ca_1.95_Eu^2+^_0.02_□_0.03_)(Si_0.94_P_0.06_)O_4_ phosphor annealed at 1473 K. The simulation was performed using the software Artemis [21] (Figure 8b), in which the Eu ion is substituted in the Ca(1n) site with 10 coordination of β-Ca_2_SiO_4_ (structural parameters by Jost et al. (1977) [18]. Figure 8c shows the calculated peaks as a sum of each peak shown in Figure 8b. With radial distance less than 2 Å, a distinct doublet peak appeared in the simulation in Figure 8c, although the corresponding peak was single for the measurement data in Figure 8a. The doping of the Eu ion in the Ca site would equalize the Ca(Eu)–O lengths, and hence we observed the single peak for the radial structure function in Figure 8a. This result agreed well with that obtained by Sato et al. (2014) [4]. For the doping of a large amount of Eu ions, the Eu ions would preferentially occupy the Ca (2n) site, leading to the change in emission color from green to yellow, and then to red, because the PL property is closely related to the crystal field of the host material. 

### 2.3. Green-Yellow-Emitting P-Doped C_2_S Phosphor with a Large Amount of Eu^2+^ Ions

**Figure 9:**
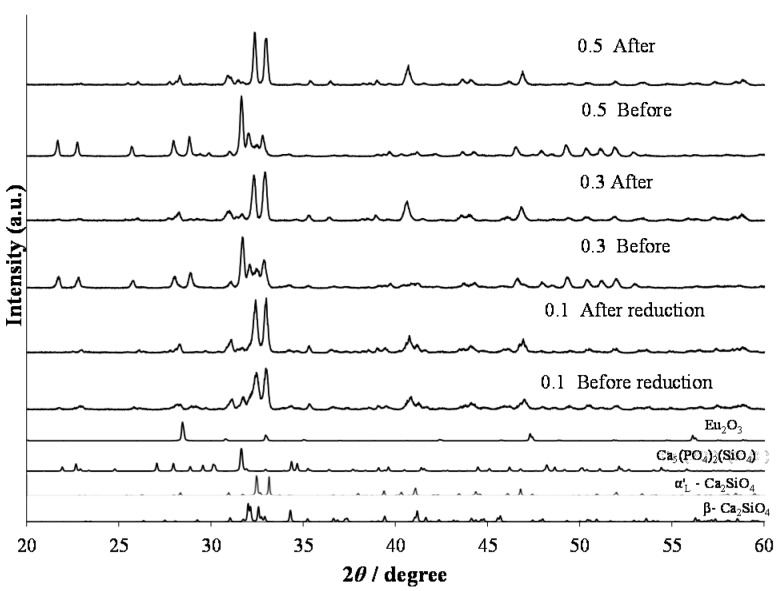
XRD patterns of (Ca_1.97–*y*_Eu^2+^*_y_*□_0.03_)(Si_0.94_P_0.06_)O_4_ phosphors with (*y*) = 0.1, 0.3, and 0.5 annealed at 1473 K and consequently reduced under a 97% Ar–3% H_2_ reductive atmosphere.

**Figure 10:**
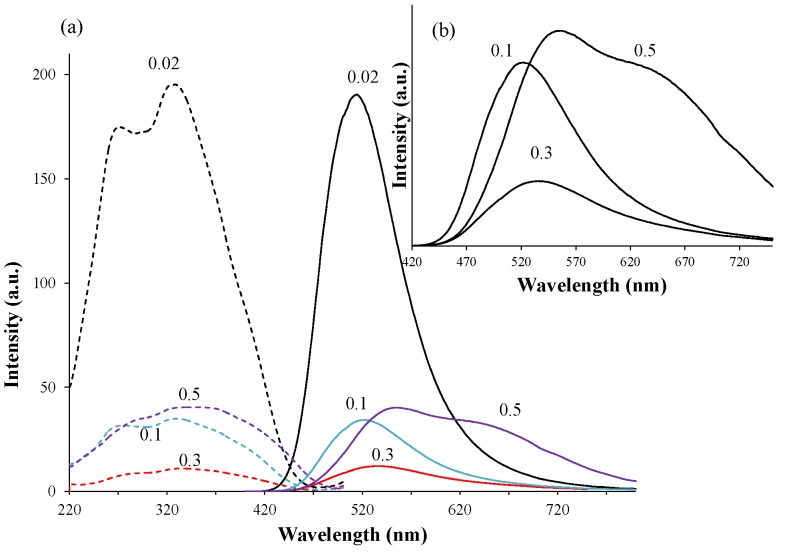
PL properties of (Ca_1.97__–*y*_Eu^2+^*_y_*□_0.03_)(Si_0.94_P_0.06_)O_4_ phosphors with (*y*) = 0.02, 0.1, 0.3, and 0.5. (**b**) is an enlarged part of the figure in (**a**).

In the formula of (Ca_1.97–*y*_Eu^2+^*_y_*□_0.03_)(Si_0.94_P_0.06_)O_4_, the phosphors were synthesized with a large Eu^2+^ content (*y* = 0.1, 0.3, and 0.5). Figure 9 shows XRD patterns of the phosphors that were annealed at 1473 K and consequently reduced under a 97% Ar–3% H_2_ reductive atmosphere. With increasing Eu content, it was obvious that the structural change occurred after the reduction process. Before the reduction treatment, the β and α’_L_ phases, together with the Ca_5_(PO_4_)_2_(SiO_4_) phase, coexisted. However, after the reduction, the main constituent phase was the α’_L_ phase. For the doping of Eu with a smaller amount, the phase compositions were almost the same between the phosphors before and after the reduction treatment, as shown in Figure 1 and Figure 4. Therefore, the phase compositions were effectively affected by the doping of a large amount of Eu^2+^, the ionic radius of which is larger than that of Eu^3+^. Figure 10 shows the PL intensities of phosphors with the Eu^2+^ content of *y* = 0.02, 0.1, 0.3, and 0.5. With increasing Eu content, the PL intensity steadily decreased due to the effect of concentration quenching. The top peaks shifted to the longer wavelength sides; they are 512 nm with *y* = 0.02, 525 nm with *y* = 0.1, and 533 nm with *y* = 0.3. With the Eu^2+^ content of *y* = 0.5, two peak-tops appeared at around 550 nm for one top and around 640 nm for the other. As described above, the emission peak wavelength decreased by the expansion of Eu–O bond lengths by the phase transition from β to α’_L_ [18]. The emission color would change in accordance with the site preference of Eu^2+^ ions between the distinct two sites of Ca(1n) and Ca(2n). Sato et al. reported that the emission color of Ca_2–*x*_Eu*_x_*SiO_4_ phosphor changed from green-yellow to deep-red with increasing Eu^2+^ content [4], which was closely related to the peculiar coordination environments of Eu^2+^ in the two Ca sites of the host C_2_S [22].

## 3. Materials and Methods 

The phosphors were synthesized by a conventional solid-state reaction without flux regents because their compositions should be precisely controlled. The starting materials used were the chemicals of CaCO_3_, SiO_2_, CaHPO_4_·2H_2_O, and Eu_2_O_3_ (>99.9% grade) for the preparation of the Eu^2+^-doped C_2_S phosphors. The chemicals were weighted in molar ratios of [Ca:Eu:Si:P] = [2−x/2−y:y:1−x:x] with 0 ≤ *x* ≤ 0.20 and 0.02 ≤ *y* ≤ 0.5*,* the chemical variations of which correspond to the general formula (Ca_2–*x*/2–y_Eu^2+^_y_□*_x_*_/2_)(Si_1–*x*_P*_x_*)O_4_. These powder specimens were thoroughly mixed with a small amount of acetone in a planetary ball mill (Pulverisette P-6, Fritsch, Dresden, Germany. The well-mixed materials were subsequently pressed into pellets, heated at 498 K for 6 h, 973 K for 2 h, and then at 1273 K for 8 h in air. The densely sintered disc-shaped specimens thus obtained were subsequently annealed for 4 h at 1373–1773 K, which correspond to the stable temperature regions of α’_L_ (1373 K), α’_H_ (1473, 1573, and 1673 K), and α (1773 K), and then cooled to ambient temperature in the electric furnace. Finally, these samples were heated at 1473 K for 3 h under a 97% Ar–3% H_2_ reductive atmosphere. Phase identification was made based on the XRD data (CuKα), which were obtained on a RINT 2500 device (Rigaku Co., Ltd., Tokyo, Japan) operated at 40 kV and 200 mA. The phase compositions were determined from the X-ray profile intensity data (CuKα_1_) collected on another diffractometer in the 2*θ* range of 25.0°–43.0° (X’Pert PRO Alpha-1, PANalytical B.V., Almelo, The Netherlands) operated at 45 kV and 40 mA. The profile intensity data were examined by the Rietveld method [23] using a computer program RIETAN-FP [24,25]. The refinement resulted in the satisfactory reliability (*R*) indices of *R*_wp_ = 4.74%, *S* (=*R*_wp_/*R*_e_) = 1.72, and *R*_p_ = 3.61%. TEM images were observed using a conventional TEM (JEM-2100F, JEOL, Tokyo, Japan) equipped with an energy-dispersive spectroscopy. Eu L3-edge X-ray absorption fine structure spectroscopy (XAFS) was measured at beam line BL5S1 in Aichi Synchrotron Center with fluorescence mode at room temperature. The simulation of XAFS was performed on the software Artemis [21]. Excitation and emission spectra were obtained using a fluorescence spectrophotometer (F-7000, HITACHI, Tokyo, Japan).

## 4. Conclusions

A series of (Ca_2–*x*/2–*y*_Eu^2+^*_y_*□*_x_*_/2_)(Si_1–*x*_P*_x_*)O_4_ (*x* = 0.06, 0.02 ≤ *y* ≤ 0.5) with various color-emitting phosphors were successfully synthesized by a solid-state reaction. We clarified the effect of heat treatment on the emission color using X-ray powder diffractometry, transmission electron microscopy, and X-ray absorption fine structure spectroscopy. The PL properties of the phosphors were closely related to the structural change from β to α’_L_, which occurred by heat treatment, and the amount of doped Eu ions.

1. With red-emitting (Ca_1.95_Eu^3+^_0.02_□_0.03_)(Si_0.94_P_0.06_)O_4+δ_ phosphor, the PL intensity increased with increasing annealing temperature, with the highest PL intensity reached when annealed at 1773 K. There was no significant change in the ratio of electric-dipole transition/magnetic-dipole transition during the annealing.
2. With green-emitting (Ca_1.95_Eu^2+^_0.02_□_0.03_)(Si_0.94_P_0.06_)O_4_ phosphor, the highest PL intensity was observed when annealed at 1473 K. Because the phase composition was both α’_L_ and β, there must be many α’_L_/β boundaries, which would provide a favorable luminescent environment of the Eu^2+^ ion in the host material. We confirmed that the Eu^2+^ ion preferentially occupied the Ca(1n) site, based on the simulation and experimental data. With increasing annealing temperature, the emission peak wavelength decreased due to the expansion of the Eu–O bond lengths.
3. With the increase of the *y*-value for (Ca_1.97–*y*_Eu^2+^*_y_*□_0.03_)(Si_0.94_P_0.06_)O_4_, the emission color accordingly changed from green (*y* = 0.02) to yellow (*y* = 0.5). This color change was caused by the increase of Eu^2+^ occupancy at the Ca(2n) site with respect to the Ca(1n) site.

## Figures and Tables

**Table 1:** Emission and excitation wavelengths of (Ca_1.95_Eu^2+^_0.02_□_0.03_)(Si_0.94_P_0.06_)O_4_ phosphors.

Annealing Temperature (K)	*λ*_ex_ (nm)	*λ*_em_ (nm)
1373	323	512
1473	324	512
1573	330	509
1673	322	497
1773	319	490

## References

[1] Choi S.-W., Hong S.-H. (2009). Characterization of Ca_2_SiO_4_: Eu^2+^ Phosphor Synthesized by Polymeric Precursor Process. J. Am. Ceram. Soc..

[2] Jang H.S., Kim H.Y., Kim Y.S., Lee H.M., Jeon D.Y. (2012). Yellow-emitting γ-Ca_2_SiO_4_:Ce^3+^, Li^+^ phosphor for solid-state lighting: luminescent properties, electronic structure and white light-emitting diode application. Opt. Express.

[3] Zhang Y., Chen J., Li Y., Seo H.J. (2014). Monitoring of hydroxyapatite conversion by luminescence intensity of Eu^3+^ ions during mineralization of Eu^3+^-doped β-Ca_2_SiO_4_. Opt. Mater..

[4] Wei F., Jia Q. (2015). Massive production of A_2_SiO_4_:Eu^3+^ and A_2_SiO_4_:Eu^2+^ (A = Ca, Sr, Ba) microspheres and luminescent properties. Superlattices Microstruct..

[5] Sato Y., Kato H., Kobayashi M., Masaki T., Yoon D.H., Kakihana M. (2014). Tailoring of deep-red luminescence in Ca_2_SiO_4_: Eu^2+^. Angew. Chem. Int. Ed..

[6] Fukuda K., Maki I., Ito S., Miyake T. (1997). Structural change in phosphorus-bearing dicalcium silicates. J. Ceram. Soc. Jpn..

[7] Furuya S., Nakano H., Yokoyama N., Banno H., Fukuda K. (2016). Enhancement of photoluminescence intensity and structural change by doping of P^5+^ ion for Ca_2–x/2_(Si_1–x_P_x_)O_4_: Eu^2+^ green phosphor. J. Alloys Compd..

[8] Taylor H.F.W. (1997). Cement Chemistry.

[9] Fukuda K. (1999). Phenomenological analysis of α_L_’-to-β martensitic transformation in phosphorus-bearing dicalcium silicate. J. Mater. Res..

[10] Fukuda K., Maki I., Ito S. (1996). Structure change in strontium oxide-doped dicalcium silicates. J. Am. Ceram. Soc..

[11] Fukuda K., Maki I., Ito S. (1996). Thermal hysteresis for the α′_L_ ↫ β transformations in strontium oxide-doped dicalcium silicates. J. Am. Ceram. Soc..

[12] Nakano H., Yokoyama N., Banno H., Fukuda K. (2016). Enhancement of PL intensity and formation of core-shell structure in annealed Ca_2–__x_/_2_(Si_1–__x_P_x_)O_4_:Eu^2+^ phosphor. Mater. Res. Bull..

[13] Fujishiro F., Murakami M., Sekimoto R., Arakawa T., Hashimoto T. (2012). Development of New Oxide Phosphors by Controlling Substitution Site of Lanthanide Ion. Nihon Daigaku Bunrigakubu Shizenkagakukenkyujo Kenkyukiyou.

[14] Nakano H., Ozono K., Hayashi H., Fujihara S. (2012). Synthesis and luminescent properties of a new Eu^3+^-doped Li_1+x_(Ta_1–z_Nb_z_)_1–x_Ti_x_O_3_ Red phosphor. J. Am. Ceram. Soc..

[15] Ichioka H., Furuya S., Asaka T., Nakano H., Fukuda K. (2015). Crystal structures and enhancement of photoluminescence intensities by effective doping for lithium tantalate phosphors. Powder Diffr..

[16] Luo Y.Y., Jo D.S., Senthil K., Tezuka S., Kakihana M., Toda K., Masaki T., Yoon D.H. (2012). Synthesis of high efficient Ca_2_SiO_4_:Eu^2+^ green emitting phosphor by a liquid phase precursor method. J. Solid State Chem..

[17] Mori K., Kiyanagi R., Yonemura M., Iwase K., Sato T., Ito K., Sugiyama M., Kamiyama T., Fukunaga T. (2006). Charge states of Ca atoms in β-dicalcium silicate. J. Solid State Chem..

[18] Jost K.H., Ziemer B., Seydel R. (1977). Redetermination of β-dicalcium silicate. Acta Crystallogr..

[19] Udagawa S., Urabe K., Yano T., Takada K., Natsume M. (1979). Studies on the Dusting of Ca_2_SiO_4_—The Crystal Structure of α’_L_-Ca_2_SiO_4_. Proc. Jpn. Cem. Eng. Assoc..

[20] Dickens B., Brown W.E. (1971). The Crystal Structure of Ca_5_(PO_4_)_2_SiO_4_ (Silieo-Carnotite). Tschermaks Miner. Petrogr. Mitt..

[21] Ravel B., Newville M. (2005). ATHENA, ARTEMIS, HEPHAESTUS: Data analysis for X-ray absorption spectroscopy using IFEFFIT. J. Synchrotron Radiat..

[22] Tezuka S., Sato Y., Komukai T., Takatsuka Y., Kato H., Kakihana M. (2013). Eu^2+^-Activated CaSrSiO_4_: a New Red-Emitting Oxide Phosphor for White-Light-Emitting Diodes. Appl. Phys. Express..

[23] Izumi F., Momma K. (2007). Three-dimensional visualization in powder diffraction. Solid State Phenom..

[24] Brindley G.W. (1945). The effect of grain or particle Size on X-ray reflections from mixed powders and alloys, considered in relation to the quantitative determination of crystalline substances by X-ray methods. Philos. Mag..

[25] Young R.A., Young R.A. (1993). Introduction to the Rietveld method. The Rietveld Method.

